# An Inquiry into the Sources and Mode of Action of the Poison of Fever

**Published:** 1841-10-01

**Authors:** Alfred Hudson

**Affiliations:** Physician to the Navan Fever Hospital


The following are the data upon which the proof of the connexion of diseased mucous follicles with the peculiar effects of a morbid poison upon the biliary excretion, may be rested.

1st. It may be considered as admitted, that the special characters of substances fitted for assimilation are absence of active chemical qualities, and the capability of yielding to transformations; and that every substance may be considered as nutriment, which loses its former properties when acted on by the vital principle, and does not exercise a chemical action upon the living organ.

2ndly. That in the progress of the functions of nutrition, certain chemical and organic substances are produced, and from time to time are present in the blood; which products it is the office of the different excreting organs to discharge from that fluid,—the relative activity of these organs depending partly upon the matter to be eliminated, and partly on other circumstances; thus we have seen that, in one situation the lungs assume a disproportionate activity, in others, the liver, &c.

The foregoing propositions being admitted, the following may be regarded as convertible from them. If substances be introduced into the blood which are *not* capable of assimilation or of affording nutriment—whether from their chemical qualities or from their condition (of decomposition)—it will follow that, instead of these suffering the transformations which food undergoes to become assimilated, the blood will undergo their transformation and disease will be produced.

Also, that numerous modifications in the composition and condition of the compounds, produced from the elements of the blood, may be the immediate result of the introduction into it of these substances, and a change in the quality of the excretions may thus be the first indication of the action of the poison, as well as of the effort made to expel it.

Numerous facts and observations tend to shew that, in the case of organic or putrid poisons, the *liver* is the organ by whose excretions an attempt is made to rid the blood of the new products thus formed in it.

As first—by a reference to the experiments of injection of putrid pus, &c., into the veins of animals, performed by Magendie, Gaspard, Cruveilhier, &c., it will be seen that when the animal recovered it was after copious discharges of a vitiated character from the bowels; to these discharges the last-named writer attributes the recovery, and adds, that it is a fundamental fact of pathology that the intestinal canal is chiefly affected in diseases caused by miasmata.

Again, if we refer to the published cases of poisoning from putrid ingesta, we see that, besides those of irritant poisoning in which the rapid rejection of the substances was followed by recovery, there is another class in which, after an interval allowing of the absorption of the poison into the circulation, a different set of symptoms followed, as in the following from Dr. Christison's work on poisons:—"A family of five persons took for dinner broth made of beef, which owing to its black colour the master of the family had previously said to his wife he thought bad and unfit for use.

In the course of some hours two boys were attacked with sickness and vomiting, but appear to have got soon well, probably from the early discharge of the poison. Next morning a washer-woman, who had dined with the family, was seized with violent *pain in the bowels, diarrhœa, racking pains, and weakness in the limbs,* and did not recover for ten days. On the evening of the second day the master of the house was similarly affected and was ill for a fortnight. And a day later, his wife was also seized with a similar disorder, preceded by soreness of the throat and tongue and difficulty of swallowing, and ending fatally in fourteen days."

It is worthy of notice that the severity of these cases was in proportion to the interval allowed for absorption of the poison—altogether their resemblance to the description of the symptoms of typhoid fever, quoted from Louis, is remarkable. If we enquire why the mucous glands of the lower portion of the ileum, are more than other parts of the intestine liable to suffer from this peculiar derangement of the biliary excretion, we shall see reason to think the cause is the same as would explain their existence in greater number there than elsewhere, and that this is probably owing to the fact of a second digestion or chymification being performed in the cæcum, during which, it is believed by some physiologists,* that the entrance to the large intestine is closed, and bile collected in the lower portion of the small intestine, which does not enter the cæcum till the secondary chymification is completed. The effect of such a retention of an acrid and depraved secretion must be obviously to produce irritation in the part subjected to its influence, and the same deranged products of secretion, continuing through much of the duration of the fever, we can account for this affection not seeming to be limited to any portion of that period, but why commencing with it,—frequently even preceding it, it ordinarily survives the continuance of the most prolonged disease.

This explanation also accounts for the disease in these glands being found farthest advanced nearest to the termination of the intestine—for perforation occurring almost invariably close to the cæcum—for the lymphatic glands of the mesentery, corresponding to the diseased follicles, becoming diseased—and for the severity of these affections bearing a direct proportion to the severity of the fever, and, as it would appear, to the amount of the poison imbibed into the system.

This view of the relation of diseased follicles to the action of the septic poison differs both from that which regards dothinenteritis as the cause of fever, and from that which assigns to it a merely secondary place in this affection. To the latter are opposed the extremely frequent, and early occurrence of abdominal symptoms (as diarrhœa) in the typhoid form of fever. With the post mortem appearances in subjects examined at an early stage ; while the former is irreconcileable with the occasional absence of the lesion, the frequent want of correspondence between its amount and the gravity and fatal result of the fever, (a correspondence which should exist if the other phenomena of fever were but the sympathies of the affection of the follicles,) with the occasional persistence of the local disease after the fever has subsided, and with the presence (almost equally frequent in typhoid fever) of other lesions which cannot be considered sympathetic of this, but must be ascribed either to the immediate operation of the poison, or to that state of the blood produced by it; such are the softening of the spleen, liver and heart, and the inflammatory affection of the brain and thoracic viscera.

These pathological changes, as well as the derangements of function constituting the *febrile state,* will probably be best explained by some such hypothesis as that advanced by Dr. Hodgkin, which supposes the febrile state to depend upon a suspension, or at least very considerable interruption of that process by which, during health, the various parts of the system are continually undergoing a change, the old materials being removed while others are substituted in their place."† This hypothesis will be found perfectly in accordance with that of the

---

* Schultz quoted by Müller.

† Lectures on Mucous Membranes, Lect. 23.

Dr. Hodgkin's hypothesis seems to explain the great difference in the fatality of fever as affecting the higher and lower classes of society, since by the mode of life of the former, more nutriment being taken into the system, and more organic matters constantly present in the blood, an arrest of the process by which they are eliminated, must naturally be followed by more complete deterioration of the mass of circulating fluid, and more serious injury to the functions and structure of the organs supplied by it.

action of a morbid poison upon the blood, since it will be the natural effect of the molecular change produced in that fluid by the decomposing particles of the poison so to modify it as to render it unfit to undergo the capillary attractions constituting the processes of interstitial absorption, nutrition, and secretion; and thus instead of Dr. Smith's formula of the order of successive derangements in fever—namely, derangement of innervation—then of circulation, and lastly of secretions and of the animal fluids; the more correct one will probably be, first the molecular change in the blood, then the suspension or modification of the interstitial processes—or change of particles—then certain derangements of innervation and of the heart's action, and the result—formed fever.

According to this view, dothinenteritis is one of the phenomena of the second stage in the action of the poison, and immediately consequent upon certain modifications of the biliary (and probably also the intestinal) secretion. Its occurrence cannot be considered *essential* to typhoid fever, as the contamination of the blood may cause the molecular changes upon which the foregoing hypothesis supposes fever to depend without this—albeit its absence in typhoid fever if very rare—while, on the other hand, it may exist without fever necessarily following, for we frequently see that, of a number of individuals exposed to the same source of miasm, some will suffer an attack of typhoid fever, while others will be affected with diarrhœa or dysentery: a fact which is explained by a reference to those experiments of Gaspard, in which the recovery of the animal after putrid injection, was attended with profuse and offensive discharges—seemingly the mode of relieving the blood from the presence of the poison.

Reference has been already made to another set of cases, in which dothinenteritis occurs without fever: those namely, in which the poison was so concentrated as to produce a rapidly fatal effect, and where examination after a diarrhœa of only a day or two shewed the same peculiar affection of the follicles as in typhoid fever; the inference from such cases taken conjointly with those of fever from the same causes without dothinenteritis, must be that this lesion is *neither cause nor effect of the fever, but a concurrent and contingent effect of the poison.* Most of the other pathological changes of fever are to be explained by the alterations in the constitution of the blood. Such is evidently the cause of the softening of the spleen so invariably present in typhoid fever, and such a little consideration will shew to be the cause of the congestive character of the typhoid inflammations: for the occurrence most likely to follow such a change in the molecular attractions of the blood as will interfere with its capillary circulation, is *stagnation in this part of the system,* of which the consequences are venous congestion, passive hæmorrhages, and the softening of parenchymatous organs. The stagnant character of the typhoid pneumonia has been remarked by many; thus Dr. Williams says, " it may be almost a question whether in these cases the local disease in the lungs is not rather a congestion of blood in an altered state than an inflammation, and it is very commonly the sequel rather than the cause of the fever,"*—an opinion which seems fully warranted by dissection, as well as consistent. Huxham, with the modifications of the physical signs in this form of disease,† was so much struck with this connexion of the local affection and diseased blood, that he compared the state of the latter in these cases to the *scorbutic habit;* and Andral countenances this analogy in the following passages. " The ataxo-adynamic fever recognises for its commencement some alteration of the blood, whether this alteration may have taken place spontaneously, and produce a sort of acute scorbutus, or it may follow the introduction of deleterious agents, as miasms, virus, matters in a state of putrefaction; these agents after having modified the composition of

---

* Article Pneumonia, Cyclop. Prac. Med.

† See my Observations on Typhoid Pneumonia, Dublin Journal, V. 7, for several dissections of this disease.

the blood come to poison the nervous centres. Then the disease is everywhere, where blood and nerves are to be found, and in every part lesions may occur which perform but a secondary part in the production of the symptoms.*

*Again*—" Congestions of the parenchymatous tissues and membranes are tolerably frequent during the course of fevers. These congestions seem to depend on the rupture of equilibrium between the globules and fibrine; they are very frequent in typhoid and typhus fevers, and small-pox; the spleen and other parenchymatous tissues are usually congested in these diseases, and a diminution of fibrine as compared with the globules (whether absolute or relative) is the alteration of the blood observed in these maladies.

" The ancients concluded from the phenomena just mentioned that in the diseases in question, the blood is altered, and that its elements have a great tendency to separate. They designated by the phrase *putridity*, that morbid condition in which the vital powers seem to yield to physical causes, and the blood becomes putrescent. Borden, whose opinions as to the nature of typhoid fever are remarkably sagacious and philosophical, does not hesitate to consider that malady as connected with a general condition of the system, which he designates by the name of *acute scurvy*. This phrase is not inaccurate, so far as regards the condition of the blood. A diminution of the quantity of the coagulable material of the blood is a general fact observed in all great febrile disturbances; thus in miasmatic fevers there is first absorption of the miasma, and immediately after, the only prominent phenomenon is an alteration of the blood. This alteration which occurs in typhoid fever is the effect of some cause as yet unknown."†

Hæmorrhage is well known to be characteristic of typhoid fever. That from the air-passages is enumerated by Chomel among the distinctive symptoms of the disease. Intestinal hæmorrhage is also a frequent and an unfavourable symptom, and indicative of a diseased state of the blood. This hæmorrhage is preceded by stagnation. The softening of viscera is always observed in conjunction with an altered and fluid state of the blood. In some descriptions of softened spleen by Andral and others, the blood contained in it is compared to the lees of wine.

### SECT. V.—*Characters of the Disease produced by the Putrid Miasm.*

It has been remarked, that a general resemblance may be traced between the disease produced by the infectious animal poison of typhus and the exanthemata; its first and most striking analogy being the almost constant presence of a peculiar eruption. Another particular in which it resembles them and differs from the disease now under consideration is the absence of any constant internal lesion; the pathology of typhus being of a functional or physiological kind, while that of endemic or typhoid fever is anatomical and precise in its nature. The distinctive characters of the two affections may be thus stated. In typhus, a poison is generated by certain changes in the fluids of the living body, which, being received into the blood of a healthy individual, has a tendency to excite in that fluid the transformations from which it has itself arisen, and by which it will be reproduced: a process during which certain phenomena occur, as that of

* Clinique Medicale Translated, p. 610. Several cases are given, in which all the symptoms of typhoid fever were produced apparently by mental and bodily depression, but after death no lesion was discovered—for a striking case of this acute scorbutus, see Dr. Law's paper before quoted at p. 19.

† Lectures on the Blood, reported in Dublin Medical Press, Aug. 11, and Provincial Medical and Surgical Journal, Aug. 21.

eruption (an effort apparently to free the system from the presence of the poison), and the conclusion of which is marked by the presence in the excretions of the material necessary for the generation anew of the disease in any person into whose blood it may be received. All these phenomena may occur without appreciable change in the structure of any organ, and in fact death may be produced without any morbid appearance beyond that degree of congestion naturally connected with the modification of its processes of nutrition and secretion.

In typhoid fever the events following the introduction of the putrefactive poison are different; it will appear upon examination into these, that no new material of reproduction is generated, that the eruption is not a true exanthema or identical with that of typhus, being later in its appearance, less constant, more scanty, consisting of successive crops rather than persistent and uniform, as in typhus. A marked modification of the molecular changes of the system occurs in this as in every variety of febrile movement, but its continuance is evidently less uniform than in typhus, being subject to alternations and remissions, at times approaching those of the periodic fevers; and the critical change attending its resolution is more gradual and liable to be less certain and complete, as well as to recur by relapse, unlike that of typhus. But the most important distinction consists in the fact that the typhoid miasm has, like other putrid poisons, a tendency to be eliminated from the system through the biliary excretion, in the course of which process a peculiar form of irritation is set up in the alimentary canal, while no such tendency can be asserted of the poison of true typhus, in the majority of cases of which the biliary excretion suffers rather a diminution than otherwise.

But it may be said it is by no means proved that the typhoid affection of Louis and Chomel is of endemial origin, and in order to establish the connexion between miasm as a cause, and fever characterised by dothinenteritis as the effect, either this must be proved, or it must be made to appear that the fevers which in our own country may be traced to this source are to be distinguished from typhus by the intestinal lesion.

With regard to the French typhoid fever, we are led to infer this conclusion from the following facts: the existence of such miasm in abundance in the fosses, wells, and river of Paris—the almost invariable occurrence of gastro-intestinal affections in those newly-arrived there—the fact that typhoid fever attacks the same class so constantly as to make a change of circumstances regarded as one of the essential causes of the disease—and lastly, the testimony of the most distinguished physicians that it is not propagated by contagion. Let the experience of our large hospitals, with reference to the infection of typhus, be compared with the following statement of Andral. "In Paris, either in the hospitals or out of them, we never recognized in this disease (dothinenteritis) the slightest appearance of a contagious character. In the hospitals we do not see it transmitted from the individual who brings it from without to those who are lying in the beds next his own; neither do we see that the patients who lie in a bed previously occupied by a person who has recovered from, or who has died of a dothinenteritis, are attacked by it; neither are the physicians or medical students who come there attacked with it, more particularly those who have had to come in contact with patients labouring under the disease. Out of the hospitals what circumstances are more favourable to contagion than those generally found combined in the case of medical students who attend their companions when affected with typhoid fever? Shut up in a room which in general is very small, they pay them the most assiduous and devoted attention night and day; if the affection were contagious almost all of them would contract it, and yet we do not remember to have seen the disease even once arise in this way in a healthy individual."*

---

* Spillan's Clinique Medicale, p. 728.

Louis does not mention contagion in his observation on the causes of the disease, but Dr. Gerrhard states that, in conversation, he informed him that he had never seen a case so communicated.

But the question may be elucidated by an examination of some of the published histories of fevers occurring in this country from exposure to endemial sources, from which it will be seen that in numerous instances these were found to be attended with the characteristic dothinenteritis of the French typhoid affection. Thus in London, after making every due allowance to the advocate of the exclusive infectiousness of typhus, we must contend that the writings of Dr. Armstrong, Dr. Southwood Smith, and others, prove the frequent occurrence of continued fever from these causes, while the treatise of Dr. Smith shews how large a proportion of the fever of London is of the intestinal kind—having all the characters, symptomatic and anatomical, of the "typhoid affection." A similar remark may be made of Dr. Tweedie's work, which contains numerous cases of dothinenteritis, while it affords strong indirect testimony to the endemial source in the statement as to the period of the year at which the disease prevailed, and its remarkable subsidence under the influence of low temperature, rain, and frost—causes which exert a precisely opposite effect upon contagious typhus.*

---

* Dr. Tweedie, who is by no means a strong advocate for the malarial origin of fever, remarks, that "cold and wet Summers are always remarked to be comparatively healthy, while disorders of the bowels in such seasons are seldom observed. The number of patients admitted into the Fever Hospital, in the Autumn months of the last three years, establish this principle. In August, September, and October, 1827, there were 205; in the same months of 1828, the numbers were 170; in the Autumn of 1829, only 94 were received. The cause of this progressive diminution is undoubtedly to be traced to the cold wet Summers of the last two seasons."

A similar remark has been made by many physicians as to Dublin; thus Dr. Percival says, "it has long been observed, that protracted dry weather is peculiarly productive of fever in Dublin; and that rainy weather agrees best with the general health of its inhabitants." And while he states that the worst forms of typhous fever prevailed at an advanced period of the *Winter*, and were characterised by cerebral congestion, he thus distinctly characterizes the endemial fever: "But the seat of peculiar congestion in the autumnal fever was the inner surface of the intestines, and sometimes the mesenteric organs. The type of this epidemic was more irregular than any other; its invasion more obscure; its progress and duration less defined. The subjects of the disease were often broken down and declining constitutions, in which the digestive organs had been long impaired, &c." Could any description more resemble that of the dothinenteritis of Louis? Dr. Davidson, who argues for the identity of the two fevers, meets the above statement of Dr. Tweedie thus:—"An opinion exactly opposed to that of Dr. Tweedie is given by Dr. Armstrong. He states, that in England, typhus is evidently favoured by a low temperature, being most prevalent in the cold seasons of Winter and Spring, generally abating or disappearing, as the heat of Summer advances, and often prevailing to a considerable degree in cold wet Autumns." This passage is extracted from his work on Typhus, in which Dr. Armstrong advocated the doctrine of contagion. At a later period of his life he taught the exclusively malarial origin of fever, and in his lectures adduces in proof of that doctrine the great prevalence of fever in London during hot seasons, and particularly during the dry hot Summer of 1818. The only mode of reconciling these opposite opinions of the *same observer*, is by supposing that he described the nature of each epidemic correctly as it was presented to him, but not being prepared to recognize a distinction in their nature, was natu-

Again, while Dr. Addison ascribes the numerous cases of synochus and typhus presented at Guy's Hospital to river malaria, Dr. Bright and Dr. Hodgkin prove the identity of many of the cases received into that institution with the disease described by Louis. Similar observations have been made in other places. In Birmingham, dothinenteritis is stated to be the constant morbid appearance of the few cases of fever which occur; and on referring to Dr. Ward's account, already quoted (p. 43), of a fever which he clearly shews to have arisen from river malaria, we find it stated that it was present in all the fatal cases.

In Dublin, Dr. Cheyne marked three periods occurring in his experience, during which the contagious typhus usually prevalent, gave way to epidemics of intestinal fever, in which evidence of malaria was frequently met with, but infection not so—the pathology was that of dothinenteritis. These observations are confirmed by others; thus, Drs. Graves and Stokes have published a number of cases of peritonitis from perforating ulcer of the ileum, occurring during one of these periods, 1826-29. Dr. Kennedy states that the glands of Peyer were found by him to be more or less diseased, in a large proportion of the cases of the same period, presenting, as he remarks, a striking contrast in this respect to the fever (contagious typhus) of 1837.* Dr. Stokes also says, "In the epidemic of 1826 and 1827, we observed the follicular ulcerations (dothinenteritis of the French) in the greater number of cases. In many instances perforation took place, and the whole group of vital and cadaveric phenomena corresponded almost exactly to the dothinenteric affection of the French authors."†

We meet with similar evidence of two fevers in Glasgow. In 1836, says Dr. Stewart, I was much struck with the simultaneous occurrence in the wards of the Glasgow Fever Hospital, of two sets of cases in which the symptoms (however little most of them might seem to differ when viewed individually) presented, when taken collectively, characters so marked as to defy misconception, and to enable the observer to form with the utmost precision the diagnosis of the nature of the disease and the lesions to be revealed by dissection. More particularly it was remarkable to observe, that while in the one disease the affection in those who presented no eruption was so slight and of so short duration as to make it very questionable whether it deserved the name of typhus, and while the fatal cases presented an abundant and generally a profuse eruption; those labouring under the other, which equally and even in a much higher proportion, went on to a fatal termination, rarely presented any, and then only a very scanty eruption. It was further remarkable, that while in the one several successive patients had either been restored to health or fallen victims to the severity of the affection, the disease under which those laboured who lay side by side with them, though characterized by much less urgent symptoms, pursued its gradual course through weeks and months consecutively, and in the majority of cases to a fatal issue. And finally it was more remarkable still, that to complete the contrast already so striking, dissection proved the existence in the one disease of most extensive local lesions, in the other, the absence of all prominent local lesion whatsoever.

Dr. Stewart adds, "that during the Summer and Autumn of 1836, the cases of typhoid fever were numerous, but from the month of November in that year, (at which time both the type and amount of typhus became more formidable) till June, 1838, not more than a dozen cases, if there were even so many, and these at long intervals, were admitted for treatment."‡

This evidence is pretty clear as to the existence of two forms of disease. As

---

rally led by the evidently non-contagious character of the last observed, to doubt the correctness of his views of the origin of the first.

* Medical Report of the Cork Street Fever Hospital.

† Lectures, Lond. Med. and Surg. Journal.

‡ On Typhus and Typhoid Fevers, Edin. Med. and Surg, Journ. No. 145.

to the causes, the highest authority in Glasgow on this subject, Dr. Cowan, writing during the period referred to, and with the cases before him, says—" many of the cases of the production and propagation of disease must be ascribed to the habits of our population, to the total want of cleanliness among the lower order of the community, to the absence of ventilation in the more densely peopled districts, and to the accumulation for weeks and months together of filth of every description in our public and private dunghills, to the over-crowded state of the lodging-houses resorted to by the lowest classes, and many other circumstances unnecessary to mention."

In Edinburgh, according to Dr. Christison, "the intestinal affection has repeatedly presented itself in groups—the *constitutio dothinenterica*, to speak in nosographical language, has repeatedly appeared and disappeared as a subordinate or intercurrent epidemic, in the course of the more general epidemic—typhus." And according to Dr. Reid, such cases occur not unfrequently in the country parts of Scotland, and are occasionally sent to the Edinburgh Infirmary.

In Liverpool we are informed they occur in an intercurrent way, as in Glasgow,* and we need only refer to the evidence before the committee on the health of towns for proof of sufficient endemial causes.

In the Navan Fever Hospital there have been for the last seven years almost always two distinct forms of fever present, one or other occasionally preponderating, so as at times nearly to exclude the other. Thus for the first three years the prominent features were pain, tenderness, and meteorism of the abdomen, diarrhœa, and not unfrequently these symptoms combined with catarrh; several cases of perforation of the ileum occurred towards the close of this period; petechiæ were not frequent and were late in their appearance, and we had few instances of communication by contagion. During the three following years a highly contagious fever prevailed, and the symptoms and treatment were completely different, delirium, subsultus, dysphagia, being the ordinary symptoms, and diarrhœa being rarely met with;—nearly every case presented the measly efflorescence, and instances of contagion were as numerous as they had been rare previously. During the present Summer the prevailing type has been the abdominal fever of the first period, and instances of typhus are infrequent, certainly not a fourth of the whole, and sent exclusively from a district in which the epidemic of last year still lingers.

In America the existence of two kinds of fever has been maintained by Dr. Jackson and Dr. Gerrhard. The former says, in his report on typhoid fever, "it is plain that there are at least two species of continued fever, both in Europe and in this country, and further researches may very possibly shew more."

Dr. Gerrhard states, that "from the information we possess we should conjecture that the two diseases (British or Irish typhus and dothinenteritis), are widely different in their symptoms, anatomical characters, treatment, and mode of transmission."

The following extracts from his able paper will shew that the two forms of fever exist in America at different periods and with distinct characters, just as in our own cities.

"Dothinenteritis is by no means a rare disease in Philadelphia, although less common than at Paris. In the essay alluded to, I established the identity of the anatomical characters and of the symptoms of the fever occurring at Philadelphia with that observed at Paris.... The typhous fever which is so common throughout the British dominions, especially in Ireland, is not attended with ulceration or other lesion of the glands of Peyer....... For a period of at least ten years, there has been no epidemic of this nature at Philadelphia. In the year 1827, a large

---

* See Dr. Lombard's Letter, Dub. Med. Journal, Vol. X.

number of Irish emigrants were ill of a typhoid fever with ulceration of the small intestines, which was probably dothinenteritis, and during several successive years there were more or less extensive epidemics of remittent and intermittent fevers occurring in the neighbourhood of the city, but not often extending into the central parts of the town. In the Winter of 1835-6, a form of fever not commonly met with at the hospitals was observed from time to time. It was characterized by pungent burning heat of the skin, dusky aspect of the countenance, subsultus, delirium, with great stupor and prostration, but there was no diarrhœa, and but few symptoms referrible to the alimentary canal. It was the disease which afterwards appeared as an epidemic............. The evidence of contagion was direct and conclusive; three of the principal nurses and about a dozen assistant nurses, besides a number of patients ill with various diseases were taken with the fever. There was only one nurse of a ward in which many of the patients were collected who escaped; but several of his assistants and patients were taken ill. The wards in which the fever-patients were placed were large and well ventilated. The contrast between the two fevers in this respect (their infectious character) is obvious......... *Season of the Year.*—The epidemic began in March and continued until August—there were a few scattering cases afterwards. The Summer was unusually cool, and the Spring and Winter cold.............. *Pathological Anatomy.*—In this large number of autopsies, amounting to about fifty, there was but in one case, and that doubtful in its diagnosis, the slightest deviation from the natural appearance of the glands of Peyer............... The fact that the morbid changes pathognomonic of dothinenteritis are not met with in the typhous fever, would of itself seem conclusive that the two diseases are no more identical than pneumonia and pleurisy. Although in some respects the two affections are analogous and even similar; the radical difference of anatomical lesions is at least as well marked as the distinction between the symptoms. It is indeed singular that there should be of late a strong tendency to confound two fevers which were regarded as entirely distinct by some of the older physicians."

In the above quotations we see strongly marked the differences of the two affections as to prevailing season—symptoms, pathology, and mode of transmission, and the similarity of each to one or other of the two forms of European fever.

Having endeavoured to collect and arrange the testimony of the best authorities as to the *sources* of the fever poison, we stop upon the threshold of the extensive inquiry into the laws which regulate the diffusion of the disease in an epidemic form.

To attempt this would require the fullest investigation into the differences and analogies of the two affections, their modes of combination in the same individual, and their occurrence in an intercurrent mode during the same epidemic period, all of which modifications of disease would be found reconcileable with the theory of two poisons; the one having its elements in the blood, and reproduced in it; the other a product of putrefactive decomposition, and not reproduced in the human body; while on the other hand Dr. Davidson's recent essay contains in itself proof that his own theory of a single typhoid poison is not tenable, since it involves the assertion of the identity of two diseases, one of which (according to him) requires to be kept up by an uninterrupted series of cases of contagion, while the other, according to the best observers, never propagates itself by contagion at all. In short, according to this doctrine, we must believe that the same poison, shall at the same time and place, and among the same collection of individuals, produce two diseases totally dissimilar in their mode of access, symptoms, pathology, treatment, and mode of transmission.

